# Carotenoids From Amazonian Fruits as Functional Pigments in Active and Intelligent Food Packaging: Materials, Stability, and Technological Performance

**DOI:** 10.1111/1750-3841.71335

**Published:** 2026-08-04

**Authors:** Rafaela Julyana Barboza Devos, Luana Regina Pereira Alves, Rômulo Alves Morais, Hermanny Matos Silva Sousa, Camila da Costa Gomes, Paulo Eduardo Peres de Sa Peixoto, Glêndara Aparecida de Souza Martins

**Affiliations:** ^1^ Graduate Program in Food Science, Department of Food Science and Technology Federal University of Tocantins (UFT) Palmas Tocantins Brazil; ^2^ Department of Food Science and Technology Federal University of Tocantins (UFT) Palmas Tocantins Brazil; ^3^ Graduate Program in Food Science Federal University of Lavras Lavras Minas Gerais Brazil; ^4^ UMET CNRS Laboratory, INRAE, UMR 8207‐UMET‐PIHM, Lille University Villeneuve d'Ascq France

**Keywords:** biodegradable polymers, biomaterials, food packaging, hybrid systems, natural pigments

## Abstract

Amazonian fruits represent an underexplored source of carotenoids with high technological, functional, and economic potential for applications in food packaging. In recent years, active and intelligent packaging systems based on biopolymers and natural pigments have emerged as effective strategies for extending shelf life, monitoring food quality, and reducing waste. This critical review analyzes the incorporation of carotenoids from Amazonian fruits, particularly buriti (*Mauritia flexuosa*) and tucumã (*Astrocaryum vulgare*), into polymeric matrices for active and intelligent food packaging. Emphasis is given to structure–function relationships, optical properties, and the challenges associated with carotenoid instability, including photo‐oxidation, isomerization, and limited compatibility with hydrophilic matrices. This review discusses advanced stabilization strategies, highlighting the roles of hydrophilic carriers and hybrid systems, including nanoemulsions, Pickering emulsions, protein–polysaccharide complexes, cyclodextrin inclusion systems, and coated liposomes. These approaches enhance pigment dispersion, color stability, antioxidant performance, and controlled release, enabling their effective use as multifunctional agents and optical indicators. Evidence demonstrates that hybrid lipid–biopolymer systems can significantly reduce carotenoid degradation, enhance UV–Vis radiation blocking, and favor colorimetric responses to deterioration‐related stimuli. Furthermore, valuing Amazonian biodiversity through fruit pulps, peels, seeds, and byproducts aligns with circular‐economy principles and supports the sustainable development of materials. Finally, the review highlights regulatory, technological, and scalability challenges and identifies future perspectives for integrating carotenoids from Amazonian fruits into next‐generation packaging systems. In summary, carotenoids from Amazonian fruits emerge as promising natural alternatives to synthetic additives, supporting sustainable development.

## 1 Introduction

Active and intelligent packaging systems developed with functional biopolymers and natural pigments have emerged as robust and sustainable strategies to extend food shelf life, enable real‐time quality monitoring, and reduce food losses across the supply chain (Azman et al. 2022; Li et al. 2022). In these systems, pigments act as colorimetric indicators that respond to physicochemical changes associated with food deterioration, such as changes in pH, accumulation of volatile basic compounds, and microbial activity, thereby enabling nondestructive, easily interpretable visual assessment of freshness (Upadhyay et al. 2024). Although synthetic dyes have been widely employed for their stability and low cost, growing concerns about their toxicological risks, migration behavior, regulatory restrictions, and environmental persistence have intensified the search for natural alternatives (Pirsa and Asadi 2021; Ndwandwe et al. 2024; Guzdemir et al. 2025). Natural pigments, including anthocyanins, betalains, carotenoids, and insect‐derived colorants, have demonstrated comparable or superior sensitivity to spoilage‐related stimuli, in addition to offering biocompatibility, biodegradability, and, in many cases, intrinsic antioxidant and antimicrobial properties, thereby enhancing the multifunctionality and sustainability of intelligent packaging systems (Morais et al. 2022; Alves Morais et al. 2024).

Although widely cultivated crops such as carrots, tomatoes, and turmeric remain the primary global sources of carotenoids, several Amazonian fruits exhibit significantly higher or comparable levels of carotenoids, revealing an underutilized technological and functional potential. Native species such as tucumã (*Astrocaryum vulgare*), buriti (*Mauritia flexuosa*), pajurá (*Couepia bracteosa*), buritirana (*Mauritiella armata*), piquiá (*Caryocar villosum*), and umari (*Poraqueiba sericea*) show high proportions of *β*‐carotene relative to total carotenoid content, ranging from 72% to 92%, values comparable to or higher than those reported for established sources such as carrots (49%–65%) (Singh and Sambyal 2022). These findings suggest that Amazonian fruits represent rich, renewable, and highly competitive matrices and potential natural sources of carotenoids for food and technological applications. Thus, by neglecting the potential of these resources, especially amid growing demand for high‐performance natural and bioactive ingredients, a strategic opportunity to boost innovation and the appreciation of Amazonian biodiversity is lost.

In this context, carotenoids extracted from Amazonian fruits stand out for their high technological and functional potential, especially when incorporated into hydrophobic polymeric matrices to develop packaging systems. Native fruits such as buriti and tucumã have been previously evaluated and have shown high concentrations of *β*‐carotene and bioactive compounds with antioxidant and antimicrobial activity (Silva et al. 2022; da Silva Sousa et al. 2025; Assis et al. 2025; de Souza et al. 2025a). These species were discussed in greater detail because they currently present the most consistent scientific evidence regarding carotenoid composition and technological applications in packaging systems among the Amazonian fruits investigated. However, the efficiency of carotenoids in active and intelligent systems depends on advanced techniques that improve the incorporation and stability of these pigments in materials (e.g., solution casting, melt extrusion, mold compression, electrospinning, photo‐grafting, inkjet printing, spray coating) (Bao et al. 2022; Zhao et al. 2024; Roy et al. 2023). Technological challenges related to thermal stability, photo‐oxidation, and limited solubility still restrict their use on an industrial scale (Deng et al. 2022; Meléndez‐Martínez et al. 2023; Yildiz et al. 2023). Encapsulation, colloidal dispersion, and coating techniques have been explored to improve the incorporation and stability of these pigments and promote better material performance in intelligent detection (Cui et al. 2022; Pinheiro Bruni et al. 2020; Drosou et al. 2022).

Thus, this review presents and analyzes the state of the art in the application of carotenoids from Amazonian fruits in polymeric matrices, with a special focus on identifying and discussing hydrophilic carriers and hybrid systems that can improve the compatibility, stability, and functionality of pigments in active and intelligent packaging. The main technological challenges and future perspectives for the use of these natural compounds in food detection and preservation systems are also discussed. This study reinforces the relevance of the approach outlined in UN Sustainable Development Goal 12.3, which aims to reduce food loss and waste by 2030 through waste recovery and the use of renewable raw materials (Ardra and Barua 2022).

## 2 Optical Properties of Carotenoids

**FIGURE 1:**
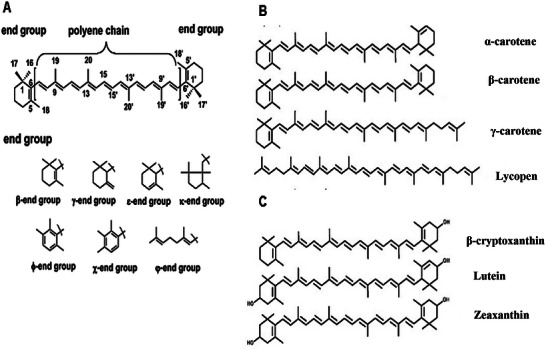
(A) Basic structures of carotenoids and their terminal groups. Structures of typical (B) carotenes and (C) xanthophylls. Adapted from Maoka (2020) and Roy et al. (2023).

Carotenoids are a class of tetraterpene pigments with 40 carbon atoms (Figure 1) that belong to the isoprenoid family, a structure composed of eight isoprene units connected in a linear, symmetrical pattern. Their basic structures generally consist of a polyene chain with conjugated double bonds and a terminal group at both ends (Figure 1A). They are divided into two groups: carotenes and xanthophylls. Carotenes, such as *α*‐carotene, *β*‐carotene, *γ*‐carotene, and lycopene, are formed only by linear hydrocarbons that can be cyclic or acyclic at the chain ends (Figure 1B). Xanthophylls, such as *β*‐cryptoxanthin, lutein, zeaxanthin, astaxanthin, fucoxanthin, and peridinin, are carotenoids that contain oxygen‐containing functional groups in their structure, such as hydroxyl, carbonyl, aldehyde, carboxylic, epoxide, and furanoxide groups (Figure 1C) (Maoka 2020).

The conjugated system of alternating single and double bonds in the center of the chain constitutes the light‐absorbing chromophore that gives the carotenoid its color. Its shades range from yellow to intense red, depending on the degree of conjugation and the presence of functional groups. Phytoene, with three conjugated double bonds, is colorless, and its conversion to phytofluene (five double bonds) generates a light‐yellow tone, followed by *ζ*‐carotene (seven double bonds) in yellow, neurosporene (nine double bonds) in orange, and lycopene (11 double bonds) in red. Thus, as the number of conjugated double bonds increases, absorption shifts to longer wavelengths, promoting the color transition from yellow to orange and finally, to red. The presence of conjugated double bonds, in addition to providing color, also stabilizes the functionality of carotenoids within the lipid membrane and proteins. The conjugated system also directly associated with bioactive properties, since compounds such as *β*‐carotene, norbixin, zeaxanthin, and lycopene exhibit strong antioxidant activity that contributes to oxidative stability and can increase the shelf life of packaged foods (Meléndez‐Martínez et al. 2023; Maoka 2020; Rostamabadi et al. 2019).

Carotenoids have been widely applied in butter and margarines, cakes, bread, dairy products, soft drinks, confectionery products, flavored milk, jams, and jellies due to their natural coloring properties and bioactive potential. However, their stability and functional performance are strongly influenced by factors such as pH, temperature, light exposure, oxygen, water activity, metal ions, and processing and storage conditions (Alizadeh‐Sani et al. 2020; Echegaray et al. 2023; Saini et al. 2022).

In the study by Tupuna‐Yerovi et al. (2023), sodium alginate films containing lycopene and *β*‐carotene showed reduced UV/visible light transmission (280–600 nm), increasing opacity and improving light barrier properties. This optical characteristic was associated with lower peroxide formation and delayed lipid oxidation in sunflower oil, demonstrating that the conjugated system contributes simultaneously to light absorption and antioxidant performance.

The high degree of unsaturation of the chain (> 9 conjugated double bonds) makes carotenoids susceptible to isomerization and oxidation, as it facilitates the delocalization of electrons from the ground state (π electrons) to an excited triplet form (π* electrons), providing a molecule with electron‐rich reactivity. These processes confer changes in color intensity by progressively shortening the conjugated double bond system. The molecule fades when exposed to heat, light, or acidic conditions, resulting in a lighter color. Under acidic conditions, protonation reactions can disrupt the conjugated double‐bond system of carotenoids, reducing molecular stability and promoting structural degradation. The extent of this process depends on acid concentration and exposure time at low pH, leading to decreased pigment stability and color intensity. Turbidity may be related to alkaline media and isomerization, which results in a spectral shift to shorter wavelengths. Exposure to these factors causes a change from the trans configuration (the molecule's more stable structure) to the cis configuration (less stable and with a less intense color). Oxidation can occur at various stages of the production chain, including extraction, storage, and incorporation into polymeric materials (Roy et al. 2023; Meléndez‐Martínez et al. 2023; Rodriguez‐Amaya et al. 2023; Kumar et al. 2024; Rostamabadi et al. 2019).

*β*‐Carotene incorporated into active packaging for fried peanuts promoted a characteristic orange coloration, increased the oxidative induction time from 4.5 to 14.1 min, and improved the oxidative stability of the material, indicating greater resistance to oxidative processes. Films containing *β*‐carotene also delayed hexanal formation during 3 months of storage at 40°C, while control films showed a 294% increase in this oxidation marker, demonstrating the relationship between carotenoid optical properties and oxidative stability in active packaging systems (Juan‐Polo et al. 2022).

Molecular aggregation also directly influences the optical and functional properties of carotenoids. Trans isomers tend to aggregate more easily than cis isomers, which makes cis isomers more soluble and more easily absorbed in organic solvents. This difference arises from the lipophilic and hydrophobic nature of these molecules. In polar media, especially aqueous media, carotenoids tend to form aggregates stabilized by dipole interactions, hydrogen bonds, van der Waals forces, and hydrophobic effects. Aggregation reduces contact with the solvent and favors the formation of poorly soluble crystals, thereby decreasing absorption and bioavailability. Heating or thermal processing partially breaks down these crystals, facilitating solubilization and increasing pigment availability. Thus, aggregation in aqueous media affects the distribution and isomerization of molecules, as well as the stability and functional performance in food systems and polymeric materials, depending on factors such as pH, presence of anions, temperature, carotenoid structure, polar substituents, and pigment concentration (Britton 2020; Rodriguez‐Amaya et al. 2023; Roy et al. 2023).

Encapsulation has emerged as an effective strategy to improve carotenoid stability and bioaccessibility during processing, storage, and food packaging applications. In this context, carotenoids extracted from Amazonian fruits have shown promising technological potential. Microcapsules from bacupari peel (*Garcinia brasiliensis*) presented high carotenoid and phenolic contents, antioxidant protection of up to 64.17%, and stable light‐yellow coloration in margarine for 30 days (de Souza et al. 2025b). Similarly, microparticles from peach palm peel (*Bactris gasipaes* Kunth) maintained a stable yellow hue to margarine during storage at 30 days, although light and room temperature promoted carotenoid degradation of up to 42.98%, confirming the sensitivity of these pigments to photo‐oxidation and thermal degradation (de Souza et al. 2025a).

These optical and aggregation properties are crucial in the formulation of active films and coatings, as they determine color stability and the efficiency of the chromatic response to environmental stimuli. However, the stability of carotenoids can be compromised by several factors, which represent a challenge for their use in food products and packaging systems. Even so, their availability in nature, health benefits, and sustainable potential have driven interest in their technological application. The Carotenoid Database, developed by Yabuzaki (2017), currently records 1204 natural compounds identified in 722 organisms, highlighting the structural and functional diversity of these pigments. Lycopene, for example, is a lipophilic red pigment predominant in red fruits and vegetables, while the orange and yellowish tones of carrots and pumpkins are due to carotenes and xanthophylls. In green vegetables, there is a higher concentration of xanthophylls and carotenoid hydrocarbons, and in marine organisms, such as salmon, the pink coloration results from astaxanthin. This variety of sources and colors reflects the biological importance of carotenoids and their potential for sustainable applications (Bufka et al. 2024).

The correlation between structure, optical absorption, and stability is essential for designing packaging systems with controlled color response. This relationship guides the development of visual pH sensors, in which carotenoid spectral modulation serves as a functional indicator of food quality. *β*‐Carotene and lutein have also been explored as natural color indicators in smart biodegradable packaging. Materials containing *β*‐carotene showed yellow‐orange coloration, where lutein imparted an intense yellow hue. Exposure to UV radiation, elevated temperature, and weathering promoted progressive discoloration, demonstrating the potential of carotenoids as visual indicators for monitoring shelf life and deterioration in intelligent packaging systems (Latos‐Brozio and Masek 2020). To synthesize the information presented, Table 1 summarizes the main traditional and Amazonian carotenoid sources, including buriti, tucumã, and other Amazonian fruits rich in individual carotenoids, highlighting their optical and technological properties, food and packaging applications, as well as the advantages and limitations associated with their use. Thus, it is possible to observe how structural variations and environmental interactions directly influence the stability and intensity of color, while also emphasizing the technological potential of Amazonian biodiversity as a source of functional pigments for active and intelligent food packaging systems.

**TABLE 1:** Properties of carotenoids from Amazonian fruits and their food applications.

Carotenoid/UV–Vis	Traditional sources	Amazonian fruit source	Classification of individual carotenoid sources^a^	Optical and technological properties	Advantages	Limitations	References
*α*‐Carotene (484 nm)	Pumpkin, carrot	Buritirana (*Mauritiella armata*)	Moderate (2.30 µg g^−1^)	Orange pulp Pulp, shells, and seeds with potential for oxidative stabilization and functional food applications	Phenolic‐rich extracts with high antioxidant potential, containing protocatechuic, vanillic, and ferulic acids. High gallic acid bioaccessibility after simulated gastrointestinal digestion in vitro.	Extraction at 61 min and 40°C reduced the relative intensity of phenolic compounds in the extracts whole without seed and pulp.	Anunciação et al. (2019) Morais et al. (2026) Morais et al. (2025) de Souza et al. (2021)
*β*‐Carotene (502 nm)	Carrot, pumpkin, green leafy vegetables, sweet potato	Buriti (*Mauritia flexuosa)*	Very high (372.00 µg g^−1^)	Orange pulp A significant increase between the *β*‐carotene results for buriti shell in natura and flour (processed at 55°C): 271.87 and 629.42 µg g^−1^, respectively	Microemulsions rich in carotenoids produced by buriti pulp and surfactant protected the carotenoids during a dynamic gastrointestinal system.	Microcapsules with buriti carotenoids with high maltodextrin content exhibit less intense red/yellow coloration and higher water content when stored at higher water activities.	de Rosso and Mercadante (2007) Sousa et al. (2024) Berni et al. (2020) Ribeiro et al. (2020)
*γ*‐Carotene (492 nm)	Tomato, papaya, citrus, crab, *Calendula arvensis*, fungi/ mushroom (Chanterelle)	Tucumã (*Astrocaryum vulgare*)	Very high (26.00 µg g^−1^)	Yellow‐orange color pulp Tucumã peels exhibit 2.1 times more total carotenoids than pulp (180.6 and 83.9 µg 100 g^−1^, respectively)	Source of pro‐vitamin A and vitamin C. Encapsulation of tucumã oil powders increases the oxidative stability under high temperature and high carotenoid retention after storage (125 days, 25°C), with controlled release of carotenoids during digestion steps.	Low antioxidant capacity in the free radical elimination by DPPH method. The acidity and peroxide index of tucumã seed oil gradually increased over the storage (amber glass, 120 days, 35°C) especially after 60 days because it presented 90.42% saturated fatty acids in their composition.	Noronha Matos et al. (2019) Barbosa‐Filho et al. (2008) da Silva Sousa et al. (2025) Santos et al. (2021) Gualberto et al. (2025)
Lycopene (522 nm)	Tomato, pink grapefruit, palm oil	Peach palm (*Bactris gasipaes*)	High (8.44 µg g^−1^)	Orange pulp Films from peach palm flour are dark, more opaque, uniform and yellowish in color, indicating they act as a barrier against UV light due to the bioactive compounds	Peach palm peel contains higher levels of carotenoids compared with pulp, classifiying it as a source of carotenoids comparable to tucumã and buriti.	Even microencapsulated carotenoids from peach palm peel exposed to light and 25°C showed high carotenoid degradation (42.98% after 30 days) due to photo‐oxidation and thermal degradation processes.	de Rosso and Mercadante (2007) Desireé Sousa da Costa et al. (2023) de Souza et al. (2025a) Sousa et al. (2024)
Lutein (508 nm)	Green leafy vegetables, orange, corn seeds, egg yolk	Camu‐camu (*Myrciaria dubia* (Kunth) McVaugh)	High (6.019 µg g^−1^)	Red pulp Carotenoids content in mature fruits (100% purple peel) decreased during storage (22°C, 70% RH, 8 days), from 300 to 133 µg 100 g^−1^. In the final storage, the immature fruits (completely green peel) presented carotenoids contents equal to the semi‐mature fruits (50% green peel and 50% purple peel).	Camu‐camu pulp applied in starch films showed radical scavenging activities against DPPH and ABTS, while the pulp microencapsulates in a soy milk beverage after 72 h of fermentation increased by 90% the antioxidant activity, due to synergistic effect of food matrices.	Carotenoids content in camu‐camu pulp and peel decrease with maturity and was lower in pulp fraction.	Tiburski et al. (2011) Zanatta and Mercadante (2007) Grigio et al. (2021) García‐Chacón et al. (2023)
*β*‐Cryptoxanthin (478 nm)	Orange, citrus, persimmon, peach, papaya fruits, capsicum pods	Taperebá (*Spondias mombin*)	High (17.08 µg g^−1^)	Yellow pulp Microencapsulation of the taperebá peels demonstrate high retention of phenolic compounds during 60 days of storage at different temperatures (4°C, 25°C, and 40°C)	Taperebá pectin was used as a forming matrix of active film with great antioxidant capacity and total phenolics was higher than açaí (*Euterpe oleracea*).	Microwave‐assisted alkaline treatment on taperebá bagasse showed loss of pigment due to strong redox potential of NaOH on polyphenol components, leading to loss of color intensity or darkening.	Tiburski et al. (2011) Oliveira Júnior et al. (2023) Oladunjoye and Eziama (2020)
Zeaxanthin (478 nm)	Corn seeds, egg yolk	Murici (*Byrsonima crassifolia* (L.) Kunth)	Low (0.519 µg g^−1^)	Yellow pulp Total carotenoid content varied during murici storage (12°C, 16 days) but zeaxanthin content increased during storage.	Total phenolic compounds of dehydrated murici was higher when compared to fresh murici but the total carotenoids did not show a significant difference, demonstrating that there were no losses from the drying process.	Phenolic compound content decreases during the murici storage because of the ripening process.	Belisário et al. (2020) de Assis et al. (2020) de Barros Vinhal et al. (2022)

Abbreviations: NaOH, sodium hydroxide; RH, relative humidity.

^a^ Classification of individual carotenoid sources by Britton and Khachik (2009): low (0–1 µg g^−1^), moderate (1–5 µg g^−1^), high (5–20 µg g^−1^), and very high sources (≥ 20 µg g^−1^).

## 3 Hydrophilic Carriers and Hybrid Systems Compatible With Carotenoids

Carotenoids constitute one of the most studied groups of natural pigments, recognized both for their visual impact, conferring vibrant colors from yellow to red, and for their biological functions, such as antioxidant activity, provitamin A action, modulation of the inflammatory response, and cellular photoprotection (Chisté et al. 2021; Soares et al. 2022). Despite their functional potential, the technological use of these compounds remains limited by their chemical and structural instability. Factors such as light, oxygen, temperature, pH, and the presence of catalytic metals promote isomerization, oxidation, and degradation reactions, resulting in loss of color, bioactivity, and bioavailability (Monteiro et al. 2021).

The intrinsic hydrophobicity of carotenoids, resulting from their long‐conjugated polyene chains and the absence of polar functional groups, restricts their dispersion in aqueous media and their incorporation into hydrophilic matrices. This characteristic poses an obstacle to its application in packaging, which is generally composed of hydrophilic natural polymers, such as polysaccharides (pectin, starch, chitosan) or proteins (gelatin, casein, vegetable proteins). The incompatibility between the pigments' lipophilic nature and the matrix's polar nature can lead to surface migration, colloidal instability, and premature degradation of the active compounds (de Souza Mesquita et al. 2020a). In this context, stabilization and compatibilization strategies have been central to enabling the application of carotenoids in aqueous systems and in active and intelligent packaging. Among these strategies, the use of hydrophilic carriers capable of forming inclusion complexes or interactive networks with pigments stands out. Another strategy is the development of hybrid systems, which integrate lipophilic and hydrophilic domains to ensure homogeneous dispersion, photochemical protection, and controlled release of carotenoids (de Souza Mesquita et al. 2019; Martínez‐Girón et al. 2025).

In addition to preserving bioactive properties, these systems confer new technological functionalities to materials, such as selective blocking of UV–Vis radiation, prolonged antioxidant capacity, and optical sensitivity to environmental changes (da Silva Sousa et al. 2025). The application of carotenoids encapsulated in biopolymeric films and coatings has enabled the development of multifunctional packaging that not only protects food from oxidation but also serves as a visual indicator of deterioration, promoting safety and transparency for consumers (de Souza Mesquita et al. 2020b; Soares et al. 2022). Therefore, understanding the stabilization mechanisms of carotenoids and their interactions with hydrophilic matrices is essential for the development of sustainable, active, and intelligent materials that integrate bioactivity, technological performance, and biodegradability. Table 2 presents the functions and technological properties of carotenoid‐based biopolymeric systems developed from Amazonian fruit sources, highlighting their potential applications in food, cosmetic, and active packaging systems.

**TABLE 2:** Functions and technological properties of carotenoid‐based biopolymeric systems from Amazonian fruit sources.

System	Polymer matrix	Carotenoid source	Extraction/incorporation strategy	Functional results	Packaging function	Application	References
Lipid‐biopolymer nanoemulsions	Coconut milk matrix + sunflower oil emulsion	Peach palm (*Bactris gasipaes*)	Ultraturrax homogenization + ultrasound‐assisted emulsification	Bioaccessibility of 29% for *α*‐carotene and 18% for *β*‐carotene	Protection of lipophilic bioactives	Medicines, vitamins, cosmetics	Martínez‐Girón et al. (2025)
Protein–polysaccharide complex	Soy protein isolate (SPI) + high‐methoxyl pectin (HMP)	Buriti oil (*Mauritia flexuosa*)	High‐pressure homogenization of oil‐in‐water emulsion stabilized by SPI–HMP electrostatic complexes	Viscoelastic gel behavior and shear‐thinning properties Stability for ≥ 7 days	Encapsulation and emulsion stability/dispersibility	Confectionery products, dairy products, sauces	Freitas et al. (2020)
Maillard‐associated pigment system	Natural fruit matrix + pectinase	Tucumã peel (*Astrocaryum vulgare*)	Enzyme‐assisted extraction + thermal/photostability evaluation	Pigment darkening via reactive carbonyl species formation and ascorbic acid degradation Higher stability under refrigeration	Color stability and degradation behavior of natural pigments	Bakery products, margarine, meat products	Miranda et al. (2024)
Pickering's emulsion	Cellulose nanofibrils (CNF)	Buriti oil (*Mauritia flexuosa*)	Oil‐in‐water Pickering emulsion stabilized by CNF	Stability for 30 days without coalescence Shear‐thinning rheological behavior	Emulsion stabilization and antimicrobial	Frozen foods, dairy products, cosmetic	da Ferreira et al. (2025)
Coated liposome	Lipid + chitosan	Pequi oil (*Caryocar brasiliense*)	Encapsulation in freeze‐dried liposomes	Antioxidant activity and stability over 10‐week storage	Antioxidant and bioactive stabilization	Cosmetics, fortified foods, functional products, and beverages	Kakuda et al. (2024) Ran et al. (2020)
Active film	Chitosan film	Peach palm (*Bactris gasipaes*)	Ultrasound‐assisted extraction Ionic liquid‐mediated recovery and incorporation into films	52% increase in ABTS UV absorption > 85%	Antioxidant and photoprotection	Preservation of fruits/ vegetables, cheeses, and meats	de Souza Mesquita et al. (2020b) Monteiro et al. (2021)

Polysaccharides are the most extensively studied group of hydrophilic carriers due to their abundance, biocompatibility, and ability to form hydrated, cross‐linked networks that trap lipophilic molecules (de Souza et al. 2025a). The structure of these polymers allows non‐covalent interactions with the polyene chains of carotenoids, forming stable physicochemical complexes (de Souza Mesquita et al. 2020a). Among the prominent examples are pectin, chitosan, and starch, which can act alone or in combination. Pectin, due to its anionic character and carboxylic groups, traps cationic carotenoids and stabilizes micelles in aqueous media. Recent studies demonstrate that carotenoid emulsions encapsulated in pectin exhibit 40%–60% lower oxidative degradation rates than free pigments (Monteiro et al. 2021). Due to its positive charge in an acidic medium, chitosan interacts with pigments and free fatty acids, forming films with high surface charge density and complementary antimicrobial properties (de Souza Mesquita et al. 2020b; Oliveira et al. 2026). In addition to its role as a structuring agent, starch also serves as a controlled‐release vehicle, modulating the diffusion of carotenoids in response to humidity and temperature. This property has been explored in sustained‐release systems, in which the oxidative half‐life of pigments can be tripled compared to free systems (Noronha Matos et al. 2019; de Souza et al. 2025b).

Water‐soluble proteins are alternative or complementary carriers to polysaccharides, primarily stabilizing interfaces in emulsified systems. Whey proteins (WPI), caseinates, and gelatin are classic examples, but in recent years, modified plant proteins (pea, soy, and fava bean) have gained prominence and are capable of stabilizing lipid droplets containing carotenoids (Monteiro et al. 2021). In systems derived from Amazonian fruits, such as peach palm, the proteins naturally present in the pulp and seed (3%–5% of the total composition) have a moderate affinity for hydrophobic pigments, contributing to the formation of protein–carotenoid complexes that increase thermal stability and reduce photodegradation (da Silva Sousa et al. 2025). When combined with polysaccharides, these proteins form soluble electrostatic complexes that function as natural microencapsulates, preserving the color and bioactivity of the pigments even after thermal processing (Li et al. 2024).

Cyclodextrins (CDs) are among the most sophisticated hydrophilic carriers, forming molecular inclusion complexes with nonpolar molecules, encapsulating them within their hydrophobic interiors and exposing the hydrophilic outer surfaces to the aqueous medium. *β*‐CD and its hydroxypropyl derivatives (HP‐*β*‐CD) have proven highly efficient in stabilizing linear carotenoids, such as *β*‐carotene and lycopene (Martínez‐Girón et al. 2025). In this study, emulsions containing peach palm peel carotenoids complexed with *β*‐CD showed a 48% increase in aqueous solubility and a 35% reduction in the photochemical degradation rate after 15 days of storage under UV light, compared to the free pigment. These results demonstrate that CDs not only increase dispersion in hydrophilic systems, but also act as protective agents against oxidation catalyzed by singlet oxygen or peroxide radicals. In addition, they are biocompatible and approved for food use, which makes them especially attractive for application in edible and biopolymeric packaging (de Souza Mesquita et al. 2020a; Martínez‐Girón et al. 2025).

**FIGURE 2:**
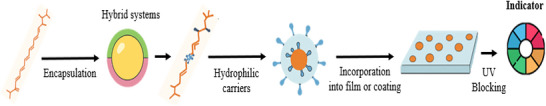
Hydrophilic carriers and hybrid systems compatible with carotenoids in active and intelligent packaging.

The antioxidant effect is amplified when carotenoids are encapsulated in hydrophilic carriers (starch, pectin, chitosan, CDs) and hybrid systems (nanoemulsions, Pickering emulsions, protein–polysaccharide complexes) (Figure 2). These systems increase the solubility, stability, and controlled release of pigments, allowing their incorporation into biopolymeric films. The resulting materials exhibit multifunctional properties, such as antioxidant activity, UV‐blocking, and colorimetric oxidation detection, contributing to the development of sustainable and intelligent packaging, as shown in Table 2. While hydrophilic carriers provide solubilization and dispersion, hybrid systems enable integration of multiple protection mechanisms, combining hydrophilic domains (pectin, starch, proteins) with lipophilic domains (vegetable oils, waxes, liposomes). This dual structure is particularly efficient for stabilizing carotenoids, as it mimics the natural microenvironment of pigments in biological matrices (de Souza Mesquita et al. 2019; Oliveira et al. 2026).

The combination of a lipid phase and hydrophilic polymers reduces pigment mobility and limits their exposure to oxygen, providing synergistic protection against oxidation and cis–trans isomerization (Monteiro et al. 2021). Furthermore, nanoemulsions of this type can be easily incorporated into starch‐ or chitosan‐based films, conferring controllable optical and bioactive properties (Oliveira et al. 2026). Starch‐ and pectin‐based films incorporated with carotenoids exhibit a UV absorption coefficient greater than 85%, providing adequate protection against photodegradation of sensitive pigments and nutrients in food (Monteiro et al. 2021). In hybrid materials, this property is enhanced. For example, Pickering emulsions stabilized by nanocellulose containing *β*‐carotene from peach palm exhibited a 60% reduction in UV transmittance (280–400 nm) and maintained 90% of the color intensity after 30 days of light exposure (Menezes Silva et al. 2023). Optical blocking not only preserves the food but also prevents the biopolymer from degrading, delaying yellowing and loss of transparency in the film (de Souza Mesquita et al. 2020b). These properties make carotenoids promising alternatives to synthetic blockers, such as titanium dioxide, whose application has been restricted due to toxicological and environmental concerns.

In chitosan films containing carotenoids extracted by ionic liquids, de Souza Mesquita et al. (2020b) observed a 52% increase in antioxidant capacity determined by the 2,2′‐azino‐bis(3‐ethylbenzothiazoline‐6‐sulfonic acid) assay (ABTS) and a 60% reduction in the peroxide index in vegetable oils stored under light for 15 days. de Souza et al. (2025a) reported that, when incorporating microcapsules of peach palm carotenoids into margarine, oxidative stability increased by 2.8 times, along with uniform coloration and absence of perceptible rancidity during 30 days of storage. These results demonstrate that the hydrophilic polymeric matrix acts synergistically with the carrier to control oxygen diffusion and favor the gradual release of active compounds. The release mechanism strongly depends on polymer–pigment interactions and environmental conditions. In starch and pectin films, release is governed by Fickian diffusion, which is proportional to the concentration and the moisture gradient. In hybrid systems, such as nanoemulsions or coated liposomes, a biphasic behavior is observed, with a rapid initial release from the surface phase, followed by sustained release due to diffusion through the matrix and slow destabilization of the interface (Martínez‐Girón et al. 2025). This controlled kinetics is essential for applications that require prolonged protection, such as foods high in lipids (butter, snacks, and processed meats).

Oil/water (o/w) nanoemulsions containing carotenoids, dispersed in a lipid phase and stabilized by hydrophilic biopolymers, are the most widely studied type of hybrid system. In a model developed by Martínez‐Girón et al. (2025), carotenoids extracted from peach palm peel were dispersed in sunflower oil and stabilized with pectin and WPI, yielding emulsions with an average particle size of 160 ± 10 nm and a zeta potential of 32 mV, indicating good electrostatic stability. After 30 days of storage, 90% retention of the initial color and 85% maintenance of antioxidant activity determined by the 2,2‐difenil‐1‐picrilhidrazila assay (DPPH) were observed, demonstrating the efficiency of the dual lipid–biopolymeric interface.

The formation of protein–polysaccharide interfacial complexes is another highly relevant strategy. These systems form via electrostatic self‐organization between positively charged (protein) and negatively charged (polysaccharide) chains, generating thick, highly hydrated interfacial layers that protect the encapsulated carotenoids. In studies with pectin and WPI, the formation of these complexes resulted in a 1.8‐fold increase in oxidative stability and better color retention (Monteiro et al. 2021). The emulsions Pickering‐based coatings, stabilized by solid particles such as nanocellulose, starch, or food‐grade silica, constitute highly resistant solid–liquid hybrid systems. In the case of peach palm, the combination of nanocellulose and native starch creates physical barriers to oxygen and improves pigment dispersion, increasing color durability and the mechanical strength of the films (Barros et al. 2024). These nanocomposites have a heterogeneous microstructure and exhibit a 35%–50% reduction in water vapor permeability and up to 60% UV blocking, critical attributes for active packaging.

Nanocellulose (CNC/cellulose nanofibrils [CNF]), derived from vegetable fibers, has also been widely used as a stabilizer in Pickering emulsions, enabling solid particles to replace conventional surfactants at the oil/water interface (Martínez‐Girón et al. 2025). In formulations containing peach palm carotenoids, CNC provides physical barriers against coalescence and oxygen diffusion, in addition to improving the mechanical and barrier properties of biopolymer films (Menezes Silva et al. 2023). Compared to conventional emulsions, Pickering systems stabilized with CNC showed a 55% reduction in *β*‐carotene degradation under light and better pigment dispersion in starch and chitosan films (Monteiro et al. 2021).

Although hybrid systems offer superior protection, studies on prolonged storage remain scarce. In lipid–biopolymer emulsions containing carotenoids from peach palm peel, a degradation constant (*k*) of 1.5 × 10^−^^3^ day^−^^1^ was observed, compared to 5.8 × 10^−^^3^ day^−^^1^ for the free pigment (Martínez‐Girón et al. 2025). These data highlight the potential for protection but also indicate the need for integrated predictive models that account for real‐world environmental variables associated with packaging and transport. According to de Souza Mesquita et al. (2020a), formulations containing peach palm carotenoids showed approximately a 2.5‐fold increase in oxidative stability and sustained release for up to 96 h using a liposome‐coated hydrophilic biopolymer strategy, simulating food storage conditions.

## 4 Composition and Carotenoids From Amazonian Fruits

### 4.1 *Mauritia flexuosa* (Buriti)

**FIGURE 3:**
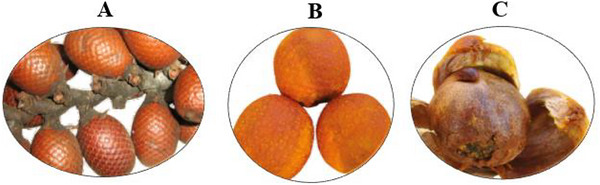
Buriti (*Mauritia flexuosa*) fruit. (A) Bunch of buriti/whole fruit. (B) Pulp. (C) Seed and peel. Adapted from Sampaio and Carrazza (2012).

The buriti (*Mauritia flexuosa*) (Figure 3) is a palm tree native to the Amazon, widely recognized for its ecological, economic, and sociocultural importance in the regions where it occurs. Also known by regional names such as *buriti‐do‐brejo*, *coqueiro‐buriti*, *itá*, *meriti*, *muriti*, and *palmeira‐dos‐brejos*, this species is adapted to humid environments and develops preferentially in flooded areas, riverbanks, and streams, where it forms dense clusters called buriti groves (Sampaio and Carrazza 2012). This palm tree, considered one of the most imposing in the biome, can reach 20–35 m in height, forming rounded crowns that are frequently used to make coverings and handicrafts. The drupe fruits measure 4–7 cm in length and 3–5 cm in diameter, with an epicarp covered in reddish‐brown scales. The mesocarp is composed of a dense, oily pulp with a yellow‐orange color (Figure 3B), a sweet‐and‐sour taste, and a high lipid content. These characteristics make buriti one of the Amazonian fruits with the highest carotenoid concentration among the fruits examined in this review (Table 3). On the other hand, to obtain viable seeds (Figure 3C), it is recommended to harvest the fruits directly from the palm tree at the beginning of natural fall or to collect them immediately after detachment (Sampaio and Carrazza 2012; Souza and Viana 2018).

**TABLE 3:** Composition of carotenoids and phenolic compounds, antioxidant capacity, and general characteristics in different parts of buriti fruit.

Buriti part	Total carotenoids	Individual carotenoid	Antioxidant capacity and total phenolics	General characteristics	References
Pulp oil	Carotenoids (1195.7 mg kg^−1^)	*β*‐Carotene (51.01 mg 100 g^−1^)	FRAP (164.86 µmol TE 100 mL^−1^) ABTS (1758.02 µmol TE 100 mL^−1^) ORAC (1.55 µmol TE 100 mL^−1^) Total phenolics (21.57 mg GAE 100 g^−1^)	*α*‐Tocopherol (263.8 mg kg^−1^) *β*‐Tocopherol (14.1 mg kg^−1^) *γ*‐Tocopherol (355.3 1 mg kg^−1^) *δ*‐Tocopherol (42.0 mg kg^−1^)	Pessôa et al. (2022) Oliveira et al. (2020) Marcelino et al. (2022)
Pulp	Total carotenoids (513.87 µg g^−1^)	*β*‐Carotene (26.7 mg g^−1^) Phytoene (0.34 µg g^−1^) All‐*trans*‐*ζ*‐carotene (0.08 µg g^−1^) All‐*trans*‐*γ*‐carotene (14.76 µg g^−1^) *Cis*‐*γ*‐carotene 2 (2.33 µg g^−1^) *Cis*‐*γ*‐carotene 3 (9.88 µg g^−1^) All‐*trans*‐*β*‐carotene (372.32 µg g^−1^) 9‐*Cis*‐*β*‐carotene (18.57 µg g^−1^) 13‐*Cis*‐*β*‐carotene (59.23 µg g^−1^) 15‐*Cis*‐*β*‐carotene (8.87 µg g^−1^) Di‐*cis*‐*β*‐carotene 2 (0.11 µg g^−1^) 5,6‐Epoxy‐*β*‐carotene (0.41 µg g^−1^) 5,8‐Epoxy‐*β*‐carotene (7.44 µg g^−1^) All‐*trans*‐*δ*‐carotene (2.09 µg g^−1^) *Cis*‐*δ*‐carotene 2 (3.67 µg g^−1^) *Cis*‐*δ*‐carotene 3 (2.42 µg g^−1^) All‐*trans*‐*α*‐carotene (3.23 µg g^−1^) Di‐*cis*‐*α*‐carotene (1.25 µg g^−1^) All‐*trans*‐lutein (0.03 µg g^−1^)	ABTS (0.84 µg TE mL^−1^) DPPH (3.18 µg g^−1^ DPPH) Total phenolics (32.1 µg GAE g^−1^)	Vitamin A (7280 RE 100 g^−1^) Gallic acid (5.06 mg 100 g^−1^) Ellagic acid (0.13 mg 100 g^−1^) Quercetin (0.77 mg 100 g^−1^) Eugenol (0.16 mg 100 g^−1^)	Leite et al. (2021) de Rosso and Mercadante (2007)
Peel	Total carotenoids (881.01 µg g^−1^)	*α*‐Carotene (182.35 µg g^−1^) *β*‐Carotene (271.87 µg g^−1^) *δ*‐Carotene (155.09 µg g^−1^) *γ*‐Carotene (144.73 µg g^−1^) Lycopene (126.96 µg g^−1^)	FRAP (568.33 mg AAE 100 g^−1^) ABTS (13.17 mmol TE g^−1^) DPPH (45.87% inhibition) Total phenolics (447.01 mg GAE 100 g^−1^)	Trigonelline (415.20 µg g^−1^) Chlorogenic (82.71 µg g^−1^) Caffeic acid (4.83 µg g^−1^) Syringic acid (14.72 µg g^−1^) *p*‐Coumaric (133.68 µg g^−1^) Ferulic acid (5.30 µg g^−1^)	Sousa et al. (2024)

Abbreviations: GAE, gallic acid equivalent; RE, retinol equivalent; TE, Trolox equivalent.

Buriti pulp presents a high caloric value (184.60 kcal 100 g^−1^) and is rich in lipids, carbohydrates, and fiber (14.28 g 100 g^−1^, 11.31 g 100 g^−1^, and 6.61 g 100 g^−1^, respectively), while protein and ash contents are low (2.42 g 100 g^−1^ and 0.93 g 100 g^−1^). Its high lipid content highlights the potential of buriti as a vegetable oil source, with oleic and palmitic acids as the predominant fatty acids (78.57 g 100 g^−1^ and 15.20 g 100 g^−1^) (Cândido and Silva 2017). Similarly, buriti peel is a fiber and carbohydrate‐rich biomass (55.50 g 100 g^−1^ and 34.31 g 100 g^−1^, respectively), with energetic value of 147.34 kcal 100 g^−1^. Maltose, xylose, and glucose were the main carbohydrates identified, while oleic and palmitic acids were also the predominant fatty acids. The peel showed low protein, lipid, and ash contents (1.79 g 100 g^−1^, 0.46 g 100 g^−1^, and 2.47 g 100 g^−1^, respectively), and nitrogen, potassium, and calcium were the main minerals detected, highlighting its potential for nutritional and technological applications (Sousa et al. 2024).

Based on the data presented in Table 3, the different fractions of buriti fruit exhibit distinct chemical and functional profiles, reflecting the biochemical complexity and technological potential of this Amazonian fruit. Cardoso et al. (2020) reported higher concentrations of vitamin C and total phenolics in buriti peel (55.22 mg 100 g^−1^ and 33.30 mg GAEq 100 g^−1^, respectively) than in the pulp (51.33 mg 100 g^−1^ and 19.31 mg GAEq 100 g^−1^, respectively), corroborating Sousa et al. (2024), who identified chlorogenic acid and trigonelline as the main bioactive compounds in the peel. In addition, ethanolic extracts showed higher antioxidant activity than aqueous extracts for both peel and pulp, reaching 190.43 and 160.11 µmol Trolox equivalent (TE) g^−1^, respectively (Cardoso et al. 2020).

The pulp oil stands out for its high content of total carotenoids, reaching values ​​close to 1195.7 mg kg^−^^1^, with a predominance of *β*‐carotene (51.01 mg 100 g^−1^), in addition to significant concentrations of tocopherols, especially the *γ*‐ (355.3 mg kg^−1^) and *α*‐tocopherol (263.8 mg kg^−1^) fractions. These results corroborate findings in the literature, which describe buriti oil as an important plant source of fat‐soluble bioactive compounds with antioxidant activity (Souza et al. 2024). The high antioxidant capacity observed in the ABTS, FRAP (ferric reducing antioxidant power), and ORAC (oxygen radical absorbance capacity) assays, reinforces the synergy between carotenoids and vitamin E, suggesting a relevant role in protection against lipid oxidation. Studies such as those by Pessôa et al. (2022) and Oliveira et al. (2020) already highlighted this behavior, associating the antioxidant profile of the oil with its oxidative stability and potential use in food, cosmetic, and pharmaceutical formulations, especially in systems where protection against free radicals is an essential functional requirement.

Recent evidence reinforces its technological potential for developing biopolymeric matrices for active, biodegradable films. Köhn et al. (2025) demonstrated that incorporating buriti oil microcapsules into sodium alginate films significantly improved functional properties, including greater elasticity and reduced water vapor permeability at low encapsulated concentrations, and conferred high oxidative stability during 12 days of sunflower oil preservation at 30°C. Complementarily, Silva et al. (2016) found that chitosan films enriched with buriti oil exhibited superior performance as an active packaging material, as evidenced by lower water solubility, greater elongation capacity, improved moisture barrier, complete inhibition of Gram‐positive and Gram‐negative microorganisms, and high biodegradability. These findings demonstrate that buriti oil is a promising ingredient for sustainable polymeric formulations, capable of combining antioxidant, antimicrobial, and mechanical properties to extend food shelf life.

Several studies have explored the potential of buriti and its derivatives across various technological and functional contexts, expanding the possibilities for the application of its bioactive compounds in food systems and polymeric materials. The fruit peel was investigated for the extraction of antioxidant compounds using pressurized fluid, an emerging technique of high efficiency, achieving significant yields of total carotenoids (23.4–1056.6 µg *β*‐carotene Eq g^−^^1^) and phenolic compounds (143–172 mg gallic acid Eq g^−^^1^) (Rudke et al. 2019). In a more applied approach, Assis et al. (2025) developed cassava starch films incorporating buriti oil (5%–10%) and evaluated their performance as active packaging materials. The film showed a protective effect against the photodegradation of *β*‐carotene, a significant reduction in UV–Vis transmittance, a high biodegradation rate (60%–70% in 15 days), and an efficient protection of oil under accelerated oxidation conditions (Resende et al. 2019). These findings highlight the remarkable phytochemical and technological potential of buriti, consolidating its importance as a strategic Amazonian resource for the development of functional foods and next‐generation sustainable packaging.

### 4.2 *Astrocaryum vulgare* (Tucumã)

**FIGURE 4:**
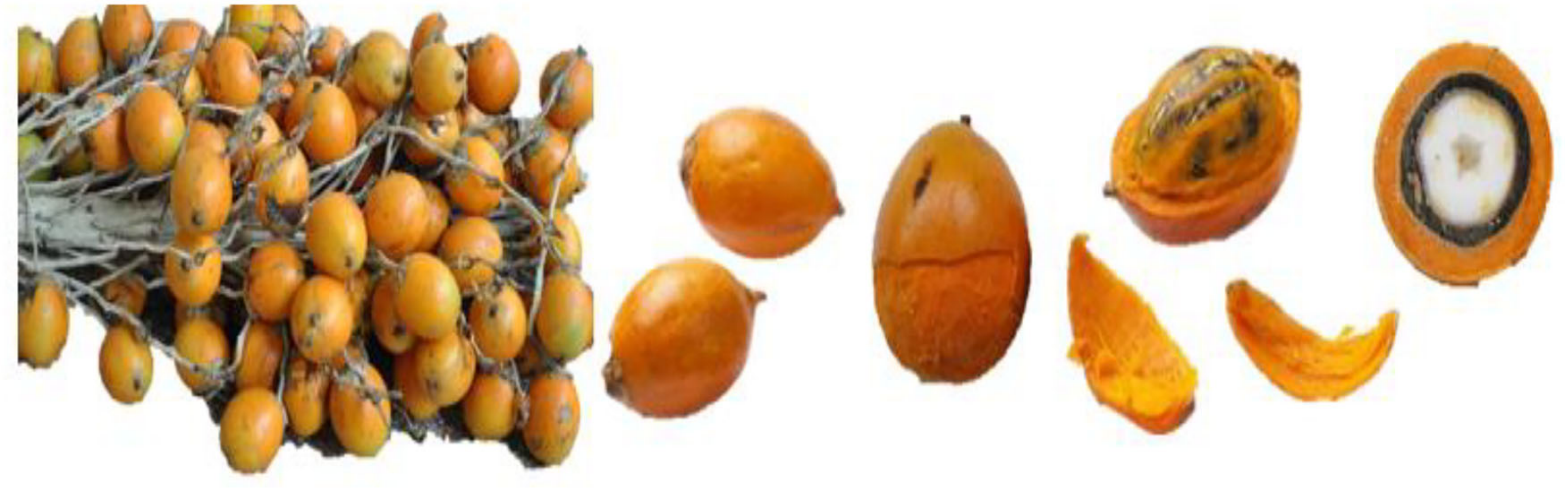
Tucumã fruit (*Astrocaryum vulgare*) and its respective parts (pulp, peel, and seeds). *Source*: Embrapa Multimedia: Image Bank.

Tucumã (*Astrocaryum vulgare*) is an Amazonian palm tree of the Arecaceae family (reaching heights of 10–25 m and diameters of 15–30 cm). Its fruits are smooth, ovoid drupes, 5–6 cm in diameter, with an average mass of 70 g per unit, and a color ranging from yellow to red. It has a fleshy, fibrous, and sweet mesocarp, while the endocarp is resistant and black, and the endosperm is homogeneous (Figure 4) (da Silva Sousa et al. 2025; Machado et al. 2022).

Lipids, carbohydrates, and fiber are the main constituents of the fruit, with respective contents of 10.87 g 100 g^−1^, 39.91 g 100 g^−1^, and 6.54 g 100 g^−1^. The carbohydrate profile highlights the presence of glucose and fructose in the fruit pulp (7.39 g L^−1^ and 6.01 g L^−1^, respectively), confirming its sweet taste, while protein and ash contents were low (3.08 g 100 g^−1^ and 2.20 g 100 g^−1^). Potassium and magnesium were the predominant minerals (70.01 mg L^−1^ and 22.50 mg L^−1^, respectively). The fruit also presented high water activity (0.91), requiring attention during storage and transport, as well as a slightly acidic (pH 6.71), titratable acidity of 1.80 g of citric acid 100 g^−1^, and high soluble solids content (9.50 °Brix), indicating potential to the agroindustrial applications without the need for citric acid addition for preservation. It is a source of vitamin C (108.31 mg of ascorbic acid 100 g^−1^), citric acid, and malic acid, with concentrations in the pulp of 310.70 mg mL^−1^ and 173.34 mg mL^−1^, respectively (da Silva Sousa et al. 2025). Its peel stands out for its high caloric content (239 kcal 100 g^−1^), due to the lipid, carbohydrate, and fiber content in its composition (7.9 g 100 g^−1^, 39.46 g 100 g^−1^, and 13.20 g 100 g^−1^, respectively) (Miranda et al. 2024). Similarly, the seeds then stand out as a raw material with high energy density (174.33 kcal 100 g^−1^) and high lipid, carbohydrate, and fiber contents (19.35 g 100 g^−1^, 34.27 g 100 g^−1^, and 29.11 g 100 g^−1^, respectively) (Gualberto et al. 2025).

The pulp also stands out for its bioactive potential. Based on the study by Hassimotto et al. (2005), the antioxidant capacity in plants and fruit pulps can be classified by the level of oxidation inhibition (IO) as high (> 70%), intermediate (40%–70%), or low (< 40%). Therefore, the total antioxidant activity of tucumã pulp can be considered high (*β*‐carotene: 87.02% inhibition of oxidation). Furthermore, according to Vasco et al. (2008), phenolic compounds in fruits and vegetables can be classified into three different categories according to their content: low (< 100 mg gallic acid equivalent [GAE] 100 g^−1^), medium (100–500 mg GAE 100 g^−1^), and high (> 500 mg GAE 100 g^−1^) for samples based on fresh matter. Thus, it is reported that tucumã falls within the medium concentration range of phenolic compounds (117.42 mg GAE 100 g^−1^ pulp), with catechin (27.08 mg mL^−1^) and gallic acid (17.01 mg mL^−1^) being the main ones present. It is estimated that the ingestion of 100 g of fresh tucumã results in a gallic acid intake similar to that from approximately 130 mL of red grape juice, which is considered one of the predominant sources of gallic acid in the human diet. The antioxidant activity evaluated by the DPPH, FRAP, and ABTS assays demonstrated the ability of tucumã pulp to neutralize free radicals, highlighting its potential benefits against oxidative stress and degenerative diseases. Although the DPPH scavenging activity was lower than that observed for peach palm, tucumã showed high antioxidant performance in the FRAP assay (105.96 µM ferrous sulfate g^−^^1^), exceeding the values reported for peach palm and bacupari. In addition, tucumã pulp exhibited high ABTS radical cation scavenging activity (591.14 µM Trolox g^−1^ pulp) (da Silva Sousa et al. 2025). Compared to peach palm peels, tucumã peels showed higher total phenolic compound content (101.68 mg GAE 100 g^−1^), although both fruits exhibited low DPPH antioxidant capacity. In the FRAP assay, tucumã peels demonstrated superior iron‐reducing antioxidant capacity (66.22 µM ferrous sulfate g^−1^) compared to bacupari and peach palm peels (4.17 and 1.25 µM ferrous sulfate g^−1^, respectively) (Gualberto et al. 2025). The antihyperglycemic and antioxidant properties of tucumã oil also highlight its potential as an alternative for treating hyperglycemia (Baldissera et al. 2017).

The carotenoid content reported in the literature highlights tucumã potential as a source of this compound. It can be used as a strategy to add value to the fruit due to its high concentration. It has been reported that tucumã contains various carotenoids, with *β*‐carotene being the main one identified. Table 4 summarizes the main carotenoids identified in other parts of tucumã, along with their bioactive content. According to Noronha Matos et al. (2019), regarding vitamin A activity, a carotenoid must possess at least one unsubstituted *β*‐ionone ring with an attached polyene side chain of at least 11 carbons. Among the identified carotenoids, *β*‐carotene and *γ*‐carotene (and their cis isomers) are the compounds that conferred vitamin A activity to tucumã peel.

**TABLE 4:** Composition of carotenoids, phenolic compounds, and antioxidant capacity in different parts of tucumã fruit.

Tucumã part	Total carotenoids (mg 100 g^−1^)	Individual carotenoid (µg g^−1^)	Vitamin A (µg RAE g^−1^)	Antioxidant capacity and total phenolics	References
Pulp	2.55	All‐*trans*‐*β*‐carotene (47.36 µg g^−1^) All‐*trans*‐*α*‐carotene (1.68 µg g^−1^) All‐*trans*‐*β*‐cryptoxanthin (1.64 µg g^−1^) 13‐*cis*‐*β*‐carotene (1.60 µg g^−1^) All‐*trans*‐*α*‐cryptoxanthin (1.30 µg g^−1^) Zeinoxanthin (1.02 µg g^−1^) All‐*trans*‐lutein (0.79 µg g^−1^) *Cis*‐*γ*‐carotene (0.89 µg g^−1^) 15‐*Cis*‐*β*‐carotene (0.80 µg g^−1^) 5,8‐Epoxy‐*β*‐carotene (0.76 µg g^−1^) *Cis*‐*β*‐zeacarotene (0.65 µg g^−1^) All‐*trans*‐*δ*‐carotene (0.60 µg g^−1^) All‐*trans*‐*β*‐zeacarotene (0.52 µg g^−1^) All‐*trans*‐*γ*‐carotene (0.36 µg g^−1^) All‐*trans*‐neoxanthin (0.35 µg g^−1^) *Cis*‐neoxanthin (0.24 µg g^−1^) *Cis*‐lutein (0.14 µg g^−1^)	4.25	FRAP (105.96 µM Fe_2_SO_4_ g^−1^) ABTS (591.14 µM TE g^−1^) DPPH (1986.27 g g^−1^ DPPH) Total phenolics (117.42 mg GAE 100 g^−1^)	da Silva Sousa et al. (2025) de Rosso and Mercadante (2007)
Pulp oil	77.82	All‐*trans*‐*β*‐carotene (212.69 µg g^−1^) 9‐*Cis*‐*β*‐carotene (51.60 µg g^−1^) 13‐*Cis*‐*β*‐carotene (39.63 µg g^−1^) 5,8‐Epoxy‐*β*‐carotene (24.12 µg g^−1^) All‐*trans*‐*α*‐carotene (16.78 µg g^−1^)	—	ABTS (2990 µM TE g^−1^) DPPH (8.59 µg mL^−1^ DPPH) Total phenolics (135.1 mg GAE 100 g^−1^)	Nascimento et al. (2021) Ferreira et al. (2021) Ferreira et al. (2022)
Peel	22.88	All‐*E*‐*β*‐carotene (78.0 µg g^−1^) All‐*E*‐*δ*‐carotene (3.0 µg g^−1^) All‐*E*‐*γ*‐carotene (26.0 µg g^−1^) *Z*‐*γ*‐carotene (8.0 µg g^−1^)	7.9	FRAP (87.56 µM Fe_2_SO_4_ g^−1^) DPPH (1402.43 g g^−1^ DPPH) Total phenolics (108.52 mg GAE 100 g^−1^)	Miranda et al. (2024) Noronha Matos et al. (2019)
Juice	—	*β*‐Carotene (67.45 µg g^−1^)	0.14	ABTS (1424 µM Trolox mL^−1^) DPPH (40.5 g mL^−1^ DPPH) Total phenolics (654.7 mg GAE 100 g^−1^)	Silva et al. (2018)

Abbreviations: GAE, gallic acid equivalent; RAE, retinol activity equivalent; TE, Trolox equivalent.

According to Kimura et al. (2007), foods containing more than 0.02 mg g^−1^ of carotenoids are considered rich sources of these compounds. Therefore, it is estimated that the intake of 100 g of tucumã is sufficient to meet the daily requirement of carotenoids (12 mg day^−1^) (Miranda et al. 2024).

According to Noronha Matos et al. (2019), the total carotenoid content in tucumã peel was approximately 2.1 times higher than in the pulp (18.06 mg 100 g^−1^ and 8.39 mg 100 g^−1^, respectively). However, the carotenoid content of tucumã pulp observed by the authors is higher than that previously reported for the same fruit (*Astrocaryum vulgare*) (7.20 mg 100 g^−1^) and for other Amazonian fruits, such as inajá (*Attalea maripa*) (0.40 mg 100 g^−1^) (Dos Santos et al. 2015), bacupari (0.14 mg 100 g^−1^), peach palm (3.47 mg 100 g^−1^) (da Silva Sousa et al. 2025), bacaba (*Oenocarpus bacaba*) (0.99 mg 100 g^−1^), and camapu (*Physalis angulata*) (1.41 mg 100 g^−1^) (Paula Filho et al. 2023). Furthermore, Dos Santos et al. (2015) reported a total carotenoid content of 4.70 mg 100 g^−1^ in buriti, highlighting it as the largest source of provitamin A in Brazil. Thus, tucumã can be considered a source of carotenoids and a precursor of vitamin A, in addition to presenting high levels of bioactive compounds in the pulp and peel, which encourages its integral use in functional foods and packaging materials (Noronha Matos et al. 2019; Oliveira et al. 2020; Machado et al. 2022).

The carotenoids extracted from tucumã peel were microencapsulated and applied to yogurt in the study by Souza et al. (2024). The authors found that microencapsulation preserved phenolic compounds and antioxidant activity, with *β*‐carotene/linoleic acid concentrations ranging from 58.45% to 62.71%. The microparticles appeared as a fine, loose, light‐colored powder due to the carrier agent's color, which diluted the pure extract's characteristic orange color. Regarding the colorimetric analysis, all values ​​of the a* parameter (ranging from green [−] to red [+]) were positive, indicating that the produced microparticles tended toward a reddish color. The b* parameter (ranging from blue [−] to yellow [+]) ranged from 14.77 to 19.23, indicating that the powders tended toward the yellow end of the scale. These results suggest the successful extraction of carotenoids from tucumã peels. When the pigmented microparticles were applied to yogurt, the color parameters showed significant differences over 30 days. The a* value indicated a change in the yogurt's hue, tending toward a redder color. The b* value also increased over the 30 days, suggesting a possible shift in the yogurt's overall color toward a more orange hue. These results highlight tucumã peel extract as a viable alternative for producing natural dyes for food applications.

Nevertheless, Bressa et al. (2021) evaluated the incorporation of tucumã oil into electrospun polycaprolactone (PCL) fibers and found promising results. In the study, tucumã oil did not affect the chemical properties of PCL, but it increased the thermal stability of the polymeric fibers and reduced the average fiber size (from 5.5 to 1.7 µm in samples loaded with tucumã oil). These findings demonstrate that tucumã oil can be incorporated into PCL via electrospinning to form fibers, without significant changes in their physicochemical properties, thereby increasing their biocompatibility. These results are promising for the development of active and intelligent food packaging systems via electrospinning, a technique of growing interest for its ability to encapsulate functional molecules directly into fibers. However, the chemical and optical properties of the loaded dyes should not be affected by their incorporation into the matrix, so as not to compromise the dyes' sensitivity to pH changes (Maftoonazad and Ramaswamy 2019).

## 5 Applications of Natural Carotenoids in Active and Intelligent Food Packaging Systems

**FIGURE 5:**
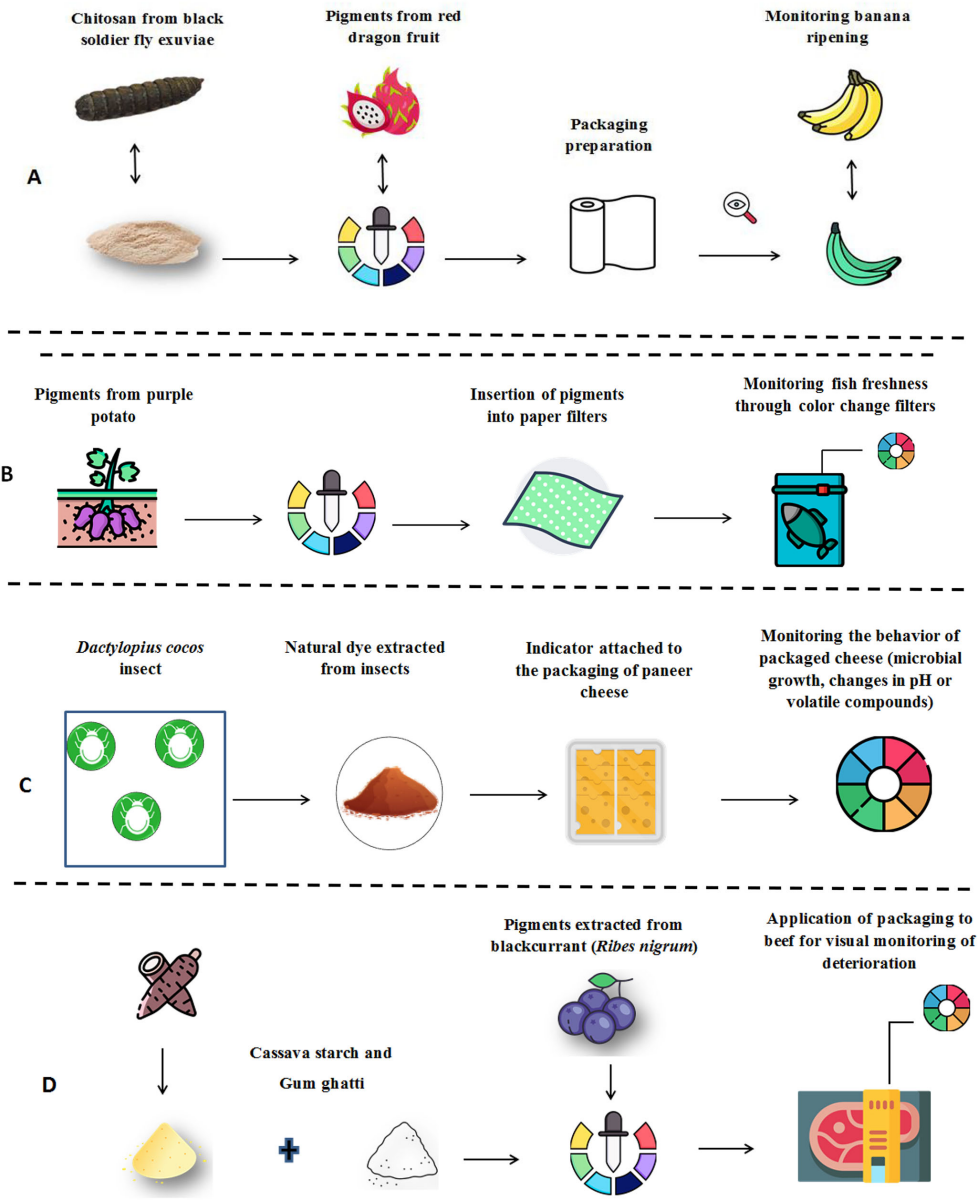
Application of active packaging in different foods. Adapted from (A) Simon et al. (2025), (B) Kim et al. (2023), (C) Sakare et al. (2024), and (D) Jiang et al. (2024).

The increasing consumer demand for safe, high‐quality, and innovative foods has driven the development of efficient and functional packaging technologies, such as active and intelligent packaging systems. In this context, natural carotenoids extracted from both established sources and Amazonian fruits have been investigated as promising bioactive pigments for food packaging applications due to their coloring properties, antioxidant activity, and sensitivity to environmental changes, allowing a comparative discussion of their technological performance in food packaging systems. Unlike conventional packaging, which serves only as a physical barrier against environmental agents, intelligent packaging incorporates sensors, indicators, and chemical, physical, or biological detection systems that respond to variations in temperature, pH, oxygen, carbon dioxide, volatile compounds, and microbial activity (Teixeira et al. 2021). These devices enable monitoring of freshness, quality, and food safety throughout the production chain, reducing losses and waste and promoting greater traceability and consumer confidence. Promising applications of these technologies have been reported across various food groups (Figure 5), highlighting their versatility and potential to optimize quality control and extend the shelf life of perishable foods.

Figure 5 presents the application of innovative, bioactive packaging based on biopolymers and natural pigments, a main trend in the sector. Figure 5A,D show the use of sustainable polymeric matrices, such as chitosan extracted from *Hermetia illucens* exuviae and cassava starch combined with ghatti gum, which exhibit recognized film‐forming properties, biodegradability, and antimicrobial potential. The incorporation of natural pigments, notably betalains, anthocyanins, and insect‐derived dyes, enables these matrices to respond colorimetrically to pH changes, the release of volatile metabolites, and redox changes, phenomena directly associated with the deterioration of foods such as bananas and beef. Given that chitosan films combined with natural pigments can exhibit detectable visual sensitivity over a pH range of 4.0–9.0, and can significantly reduce surface microbial load, they simultaneously enhance product safety and quality traceability during storage (Jiang et al. 2024; Simon et al. 2025).

On the other hand, Figure 5B,C stand out for their use of indicator systems decoupled from the packaging matrix, such as paper filters impregnated with purple potato extracts or dyes derived from *Dactylopius coccus*, used to monitor the freshness of fish and cheeses. These systems exploit the high structural instability of anthocyanins in response to pH changes and the presence of volatile amines, resulting from microbial and enzymatic activity during protein deterioration. It has been reported that anthocyanin‐based colorimetric indicators can detect biogenic amine concentrations below 10 mg N 100 g^−1^, a critical value for the sensory acceptability of fish, in addition to correlating strongly with traditional microbiological parameters, such as total mesophilic and psychrotrophic counts (Kim et al. 2023; Sakare et al. 2024). Thus, the integration of natural pigments, biopolymers, and visual indicator systems represents a promising approach to replace conventional analytical methods, offering real‐time monitoring, reducing food waste, and aligning with the principles of circular economy and sustainability, aspects increasingly demanded by industry and consumers.

The application of intelligent packaging systems is strongly influenced by several intrinsic and extrinsic parameters of the food, as well as by the packaging and the incorporated sensor. Among the intrinsic factors, the initial microbial load, moisture content, pH, oxygen, and carbon dioxide activity in the internal environment, and volatile compounds that accelerate food spoilage stand out (Chen et al. 2020; Teixeira et al. 2021; Lima et al. 2025). These variables influence both the detection mechanisms for key substances, such as amines, carbon dioxide, and molds, and the dynamics of food quality changes (Heo and Lim 2024; Ma et al. 2022). In the context of extrinsic factors, variables such as temperature and humidity during storage and variations in the logistics chain environment, such as thermal shocks, cold breaks, and packaging integrity, directly affect the proper functioning of freshness indicators, time–temperature indicators, and the overall performance of innovative packaging (Forsido et al. 2021).

Another essential aspect of innovative packaging design is the appropriate selection of manufacturing materials, as the interaction between the polymer matrix and the sensor or indicator technology determines chemical compatibility, long‐term stability, and the risk of component migration into the food. Sensors based on natural dyes, for example, must maintain a selective response to spoilage gases even under adverse conditions of humidity and light exposure, which represents a challenge for photosensitive substances (Baghi et al. 2022; Kumari et al. 2024). According to Karimi Sani et al. (2025), the use of films with high light‐ and moisture‐barrier properties facilitates the incorporation and performance of natural‐origin active sensors. In addition, the migration of substances and the functional efficiency of the packaging depend directly on the material's structural properties, since morphology, thickness, number of layers, and the polymer's chemical composition control the diffusion and permeability coefficients, thereby influencing the release of active compounds and the overall stability of the system. These challenges become even more evident when the food has high moisture or variable pH (Zubair et al. 2025; Zhang et al. 2025).

In the broader international context, the *Codex Alimentarius*, a joint body of the FAO and WHO, serves as an international reference for food safety standards and materials in contact with food, although there is currently no specific standard for active or intelligent packaging. The Codex's general guidelines, especially the General Standard for Contaminants and Toxins in Food and Feed (CODEX STAN 193–1995) and the Guidelines on Packaging for Food Products (CAC/RCP 77–2011), establish that any material intended for contact with food must be chemically inert, safe, and not transfer substances that may pose a health risk or alter the sensory characteristics of the product. In 2024, through Circular Letter CL 2024/20‐CAC, the Codex reinforced the need to advance global harmonization of guidelines for innovative materials, including recycled, bioactive, and sensor‐equipped packaging, recognizing regulatory gaps and the importance of standardized migration and toxicity data.

In addition, recent analyses indicate that the European regulatory framework imposes rigorous requirements regarding migration dossiers, traceability, and chemical characterization, which may hinder the approval of compounds of natural origin that are still poorly studied (Bokor 2025). On the other hand, in developing countries such as Tunisia, Kenya, Uganda, and Tanzania, although policies exist to reduce plastic use, gaps persist in regulatory infrastructure and technical capacity to validate alternative materials, which limit their commercial adoption (Schouten et al. 2025). Thus, the consolidation of technologies based on Amazonian pigments requires not only proof of technical and economic viability but also alignment with regulatory standards, proven toxicological safety, and regulatory transparency throughout the entire production chain.

From an economic and production perspective, the transition to biopolymers and natural‐origin active materials still faces significant barriers related to cost and industrial scale. Recent studies indicate that biopolymers have a production cost between 20% and 75% higher than that of conventional petroleum‐based polymers, mainly due to the price of raw materials, the need for high‐purity encapsulants, and associated technological processes, such as spray‐drying and lyophilization, used to stabilize pigments and natural biosensors. Despite this difference, life cycle assessment (LCA) analyses point to a trend of gradual cost reduction, driven by economies of scale, optimization of production chains, and the integration of agro‐industrial co‐products as alternative sources of biopolymers and bioactive compounds (Edo et al. 2025).

Sectoral data from the European Bioplastics Association (EUBP 2024) show that in 2024, the average utilization of industrial bioplastic capacity in Europe was approximately 58%, highlighting the challenge of scaling up production at an economically competitive rate. Even so, global projections indicate annual growth of 9%–12% for the bio‐packaging market through 2030, with a focus on sustainable, innovative packaging applications (EUBP 2024; Mordor Intelligence 2025). The application of natural pigments extracted from Amazonian fruits in food packaging represents an innovative technology at the intersection of bioeconomy and sustainability. Although natural colorants derived from red cabbage, beetroot, and grapes are already established, Amazonian fruits have emerged as promising alternative sources due to their chemical diversity and multifunctional properties (Petkovska et al. 2024; Abdulhameed et al. 2024; Sharma et al. 2025). The biodiversity of the Amazonian biome provides pigments such as anthocyanins, carotenoids, betalains, and other phytochemicals (Silveira et al. 2023; Rocha et al. 2025; Soares dos Santos Rolim et al. 2024), which contribute to a broader color range and functional properties, including antioxidant activity and responses as pH and deterioration indicators.

Recent studies on incorporating these natural pigments into polymeric matrices are available in the literature. In a survey conducted by Assis et al. (2025), oils extracted from pequi (*Caryocar brasiliense*) and buriti, rich in carotenoids, were incorporated into active packaging based on cassava starch. The authors observed that films containing buriti oil exhibited better light‐barrier performance and a lower rate of *β*‐carotene degradation under UV light, highlighting the photoprotective effect of this carotenoid derived from Amazonian biomass. Anjos et al. (2023) developed gelatin‐based films incorporating buriti oil, with the aim of applying these active packaging systems to preserve artisanal curd cheese. The results showed that the films exhibited functional activity, particularly antioxidant and antimicrobial activity in packaged cheese during storage, attributable to the presence of *β*‐carotene and tocopherols in buriti oil. These findings demonstrate the potential of carotenoids from Amazonian fruits as functional alternatives to conventional natural pigments in active packaging systems.

The literature also mentions lesser‐known Amazonian fruits, such as camu‐camu (*Myrciaria dubia* (Kunth) McVaugh) and peach palm, as sources of these pigments. Camu‐camu extract, rich in phenolic compounds and anthocyanins, enhanced the antioxidant activity of films made with teff starch, while also affecting mechanical and barrier properties (Ju and Song 2019). Desireé Sousa da Costa et al. (2023) evaluated biodegradable films formulated from peach palm fruit flour combined with chitosan and glycerol, examining physicochemical, mechanical, barrier, optical, antioxidant activity, and soil biodegradability properties. The authors observed that the yellowish color of the films derives from the carotenoids present in peach palm flour, and that higher flour concentrations resulted in more opaque films with higher phenolic compound content and antioxidant activity. Furthermore, after 15 days of soil exposure, the film lost approximately 30% of its initial mass, demonstrating biodegradability relevant to sustainable packaging applications. These studies reinforce the technological relevance of Amazonian fruits as sustainable sources for active and intelligent packaging systems, as their carotenoids and other bioactive compounds contribute to coloration, antioxidant activity, and functional and protective properties of the materials.

## 6 Final Considerations and Future Perspectives

Applying carotenoids from Amazonian fruits to active and intelligent packaging systems represents a significant advance at the interface of biotechnology, materials science, and sustainability. The results gathered in this review show that carotenoids extracted from buriti (*Mauritia flexuosa*) and tucumã (*Astrocaryum vulgare*) have functional potential, both for the stability they confer to the systems and for their antioxidant and photoprotective capacities. Recent advances demonstrate that it is possible to make highly hydrophobic compounds compatible with natural polymeric matrices while maintaining their stability and functionality. The use of natural polymeric matrices, such as starch‐ and chitosan‐based films, combined with carotenoid‐rich extracts or oils from Amazonian fruits, enhances the valorization of regional biodiversity and enables the development of materials with high added value and low environmental impact. Buriti oil in starch films resulted in a high biodegradation rate and a significant reduction in UV–Vis transmittance, while tucumã oil in electrospun fibers increased thermal stability and reduced the average diameter without compromising its chemical properties. However, consolidating these technological advances requires not only improving the techniques for incorporating and stabilizing pigments but also overcoming economic and production challenges.

Given that controlled release strategies can improve the stability of carotenoids, future research should focus on enhancing the interfacial compatibility between hydrophobic carotenoids and hydrophilic biopolymer matrices for the development of homogeneous and functionally stable films. Studies addressing pilot‐scale production, including scalability, cost‐effectiveness, regulatory aspects, and industrial applicability, are still needed to support the commercial implementation of these materials. In addition, future investigations should evaluate their toxicological safety and long‐term stability under real food storage conditions. For the industrial application of these materials in food packaging, compliance with the applicable regulatory requirements established by the relevant authorities should also be considered, particularly for active packaging systems containing bioactive compounds.

## Author Contributions

**Rafaela Julyana Barboza Devos**: conceptualization, investigation, methodology, writing – review and editing. **Luana Regina Pereira Alves**: conceptualization, investigation, methodology, writing – original draft. **Rômulo Alves Morais**: conceptualization, investigation, methodology, supervision, writing – original draft, writing – review and editing. **Hermanny Matos Silva Sousa**: conceptualization, investigation, methodology, writing – original draft. **Camila da Costa Gomes**: conceptualization, investigation, methodology, writing – original draft. **Paulo Eduardo Peres de Sa Peixoto Junior**: supervision, resources, writing – original draft, writing – review and editing. **Glêndara Aparecida de Souza Martins**: supervision, resources, writing – original draft, writing – review and editing.

## Funding

This work was partially funded by the Coordination for the Improvement of Higher Education Personnel (CAPES/Brazil) and the Conselho Nacional de Desenvolvimento Científico e Tecnológico (CNPq), for the financial support essential to project execution. G.A.S. Martins received a grant and thanks the CAPES/Brazil (n° 88881.200497/2018‐01) and PROCAD‐AM (1707/2018). G.A.S. Martins received funding thanks to the CNPq Edital de Produtividade em Desenvolvimento Tecnológico e Extensão Inovadora n° 304505/2022‐6, CAPES—Process n° 23038.000878/2021‐56, and Edital CAPES n° 018/2020 – Programa de Desenvolvimento da Pós‐Graduação—Parcerias Estratégicas nos Estados.

## Conflicts of Interest

The authors declare no conflicts of interest.
